# Developing Precision Stroke Imaging

**DOI:** 10.3389/fneur.2014.00029

**Published:** 2014-03-24

**Authors:** Edward Feldmann, David S. Liebeskind

**Affiliations:** ^1^Tufts Medical Center, Boston, MA, USA; ^2^University of California Los Angeles Stroke Center, Los Angeles, CA, USA

**Keywords:** stroke, neuroimaging, precision medicine

## Abstract

Stroke experts stand at the cusp of a unique opportunity to advance the care of patients with cerebrovascular disorders across the globe through improved imaging approaches. NIH initiatives including the Stroke Progress Review Group promotion of imaging in stroke research and the newly established NINDS Stroke Trials network converge with the rapidly evolving concept of precision medicine. Precision stroke imaging portends the coming shift to individualized approaches to cerebrovascular disorders where big data may be leveraged to identify and manage stroke risk with specific treatments utilizing an improved neuroimaging infrastructure, data collection, and analysis. We outline key aspects of the stroke imaging field where precision medicine may rapidly transform the care of stroke patients in the next few years.

Stroke imaging is a broad term that may refer to structural or functional information. Prior and current Stroke Progress Review Group (SPRG) reports promote imaging in stroke research, from the areas of prevention to treatment and recovery (1). Imaging has always been an essential component in the evaluation and management of stroke, with routine acquisition of neuroimaging studies in any patient with a cerebrovascular disorder.

Stroke advances often follow, with some delay, those made in the coronary artery disease field, which has taken bold steps to move from imaging of anatomy to imaging of function and physiology to identify disease and patients at risk. Such advances in cardiology serve as an example of precision imaging, where routine diagnostic studies may be used to cull specific information about an individual case where anatomy does not suffice. Remarkable advances have been made with the development and utilization of fractional flow reserve (FFR), an index that measures a pressure gradient across stenoses to identify coronary lesions of hemodynamic significance (2, 3). FFR identifies ischemic risk more effectively than percent stenosis of an artery and can also be identified non-invasively with conventional CT techniques. FFR is being used to identify the riskiest coronary lesions for percutaneous intervention, whether the stenosis is severe or moderate by anatomic measures. This approach has improved outcomes and lowered costs while resulting in fewer interventional procedures.

Existing and newly proposed NIH networks make imaging an important topic to consider before the next decade of stroke research efforts are launched. The newly established NINDS Stroke Trials Network will develop and conduct high-quality, multi-site phase 1, 2, and 3 clinical trials focused on key interventions in stroke prevention, treatment, and recovery (4).

The organization of the NINDS Stroke Trials network and the SPRG focus on imaging provide an opportunity to reconsider how we approach imaging in order to distil the maximum useful information from ongoing and future trials. These challenges and opportunities are in keeping with current trends to make medical care more individualized, personalized, or “precise (5):”

*“*… *the fundamental idea behind personalized medicine: coupling established clinical–pathological indexes with state-of-the-art molecular profiling to create diagnostic, prognostic, and therapeutic strategies precisely tailored to each patient’s requirements – hence the term “precision medicine.” Recent biotechnological advances have led to an explosion of disease-relevant molecular information, with the potential for greatly advancing patient care.”*

The concept of “molecular profiles” could readily be replaced with “imaging profiles”. Current catchphrases such as “creative destruction” and “team science” or “crowdsourcing” are particularly apt when considering a future of personalized or precision imaging in stroke. The traditional framework of clinical trials must be changed to achieve this vision, with an emphasis on more highly selected, homogeneous patients. Data must be made more readily available from more open sources, dramatically increasing the size of the available databases for analysis.

Realizing such a future will require a change of perspective, infrastructure, and methods for data collection and analyses. These merging influences and trends foretell a dramatic change in how stroke experts may translate their research to more effective care of their patients. We focus here on such a potential transformation of imaging research in cerebrovascular disorders, outlining the following key areas.

## Imaging Data Acquisition and Analysis Needs to be Made a Central Feature of Trial Design, Requiring Dedicated Infrastructure, Specifically Disentangled from a Focus Solely on the Investigational Treatment Being Studied in a Given Clinical Trial

Too often, imaging data are merely inclusion criteria, secondary aims are not included and collected in clinical trials. In a hypothetical treatment trial, if all patients are enrolled because they harbor a specific imaging finding, that trial cannot determine whether the imaging approach that detected that finding was beneficial, as there is no comparison group. For example, the WASID trial compared aspirin versus warfarin for treatment of 50–99% intracranial stenosis and found that aspirin was the superior treatment (6). All patients enrolled had angiographic 50–99% intracranial stenosis. The trial results were enormously useful to clinicians and researchers, but such a design cannot determine the value of using angiographic 50–99% stenosis as an imaging marker for identifying patients for treatment compared to other methods of identifying patients for treatment.

**Figure 1:**
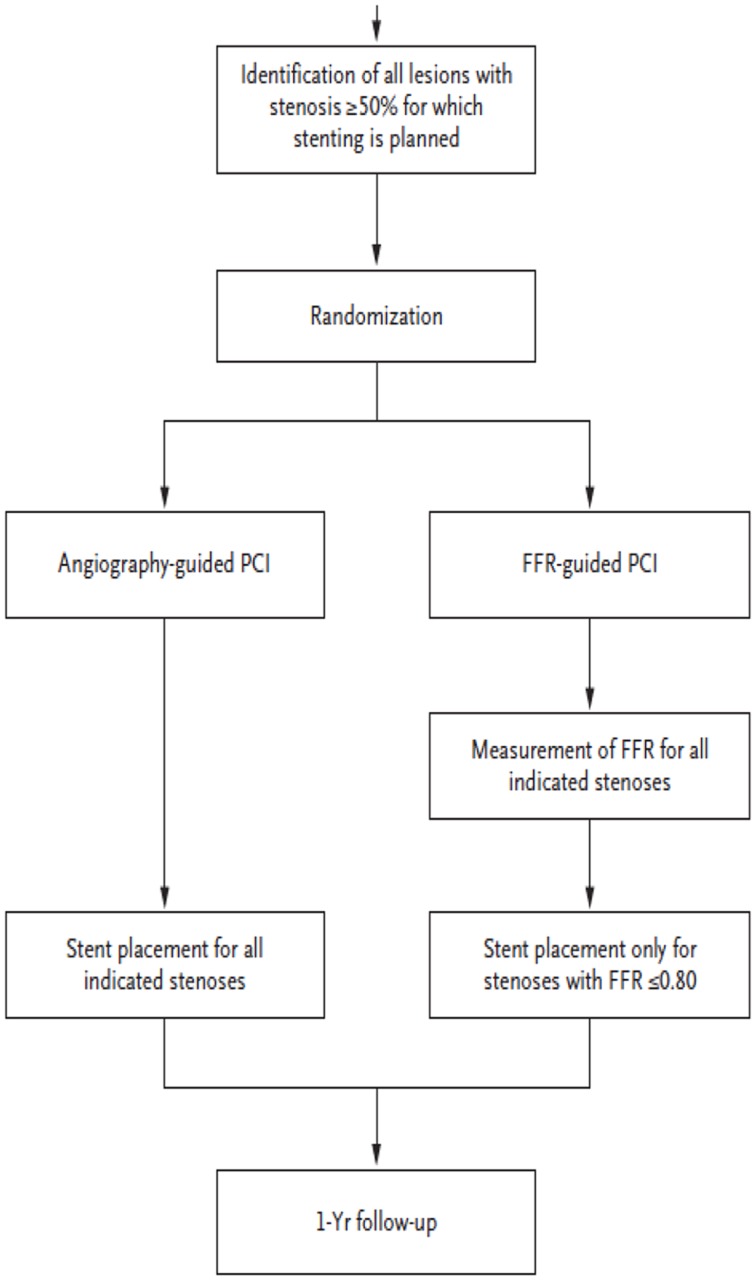
**Study design of the FAME trial**.

Contrast that situation with the designs of MR-RESCUE (7) in stroke, or FAME (2) and FAME II (3) in the study of coronary disease. In these trials, patients were tested with different imaging paradigms or enrolled with variable imaging findings, shedding light on the utility of that imaging approach for identifying subjects for a specific treatment. Figure 1 diagrams the study design of the FAME trial (2). Patients were randomized to different imaging approaches prior to treatment, yielding information on the benefit of both treatment and the imaging approach.

The FAME and FAME II trials of coronary ischemia have used advanced imaging of FFR to better distinguish ischemia causing lesions from non-ischemia causing lesions. Their results show that this imaging approach makes treatment more effective and efficient compared with an anatomic approach focused on percent stenosis. The development of fractional flow methods for intracranial atherosclerosis now heralds a similar promise for stroke, as illustrated below. In all cases, trial design and infrastructure need to carefully consider the nature and quality of imaging data to be collected.

## Imaging Data may Elucidate Pathophysiology; Should This be the Primary Aim of Some Trials, with Treatment Effect a Secondary Aim Addressing a Specific Pathophysiology?

**Figure 2:**
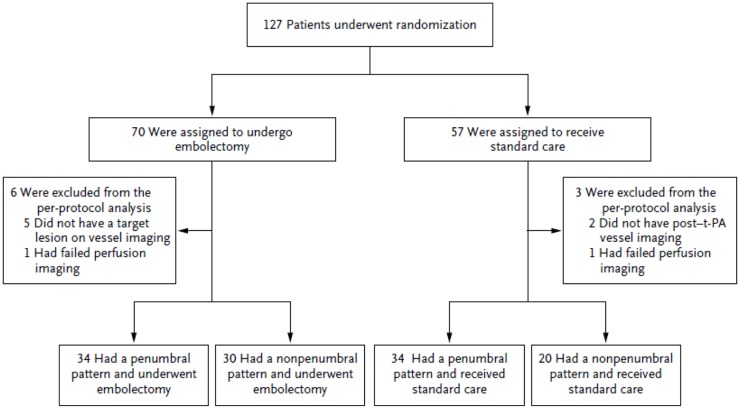
**Study design of MR-RESCUE**.

Trialists often conclude that clinical improvement is driven by treatment, but the results of MR-RESCUE show that outcomes may be better predicted by baseline imaging findings of pathophysiologic states. The Figure 2 illustrates the study design of MR-RESCUE (7), a trial that hypothesized that a favorable penumbral pattern in acute stroke predicted a differential response to thrombectomy versus standard care.

In this study, patients with a favorable penumbral pattern had improved outcomes, smaller infarct volumes, and attenuated infarct growth, as compared with patients with a non-penumbral pattern, regardless of treatment assignment.

Without a careful emphasis on pathophysiology, patients in trials may be more heterogeneous than we admit. Consider acute stroke revascularization trials: are patients with complete occlusion and partial occlusion identical? Are patients with pure clot the same as patients with atherosclerotic stenosis plus clot, with or without collaterals? These questions may be addressed by the acquisition and analysis of imaging data taken at a single time point in the care of a stroke patient. Serial imaging may also play a useful role, shedding light on the recovery phase and its mechanisms in patients with acute stroke, separate from treatment, as in newly developed approaches for assessing collateral circulation (8–12).

## Can We Discard Inflexible or Outdated Paradigms?

**Figure 3:**
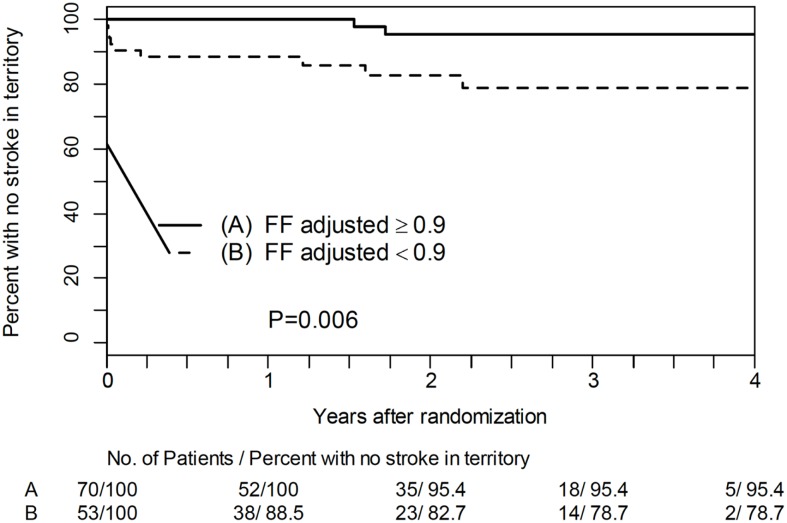
**Kaplan–Meier plot for patients with <70% stenosis surviving without recurrent stroke in the territory as a function of fractional flow assessed non-invasively with TOF-MRA**.

Treatment trials may fail if patients are suboptimally selected, as the treatment may benefit only a few, making it appear that the treatment is not effective. However, treatment may be highly effective in a different population or in selected individuals. For example, the series of WASID (6) and SAMMPRIS (13) trials of ICAD placed an imaging emphasis on structure (percent stenosis) rather than function (ischemia) in selecting eligible subjects. In order to identify the subgroup with the highest apparent risk for recurrent stroke in the territory, patients with >70% stenosis, the size of the eligible study population continually shrinks. However, nearly half of recurrent strokes occur in patients with 40–69% stenosis (14). These patients are not considered for aggressive trials. An imaging approach that uses fractional flow assessed non-invasively with TOF-MRA or CTA, rather than percent stenosis, to identify risk suggests that individuals with less severe stenosis may also harbor a high risk of recurrent stroke. Figure 3 illustrates the Kaplan–Meier plot for patients with <70% stenosis surviving without recurrent stroke in the territory as a function of fractional flow assessed non-invasively with TOF-MRA. Those with normal fractional flow ≥0.9 have a much better prognosis (15).

Thus, a new non-invasive imaging paradigm, fractional flow on TOF-MRA, may address a disease such as ICAD in a broader population of patients and more effectively identify specific individuals at high risk.

Current trials, especially those based outside of the USA, arrange trial design, cost considerations, and inclusion criteria to answer a pragmatic question: “does treatment X help patients with Y?” This is indeed a valuable, practical question. The difficulty is that these questions are posed with the supposition that patients who appear clinically similar, fitting all inclusion criteria, actually are similar. The point the authors wish to make herein is that without more precise pathophysiological data, especially imaging data, this basic assumption is flawed and contributes to waste in trial design and execution. Collection of substantial amounts of imaging data on a routine basis in large trials will facilitate the analyses required to improve the selection of patients for future studies.

## Precision Stroke Imaging Will Require Advances in Analytic Techniques, Not Necessarily New Imaging Modalities

**Figure 4:**
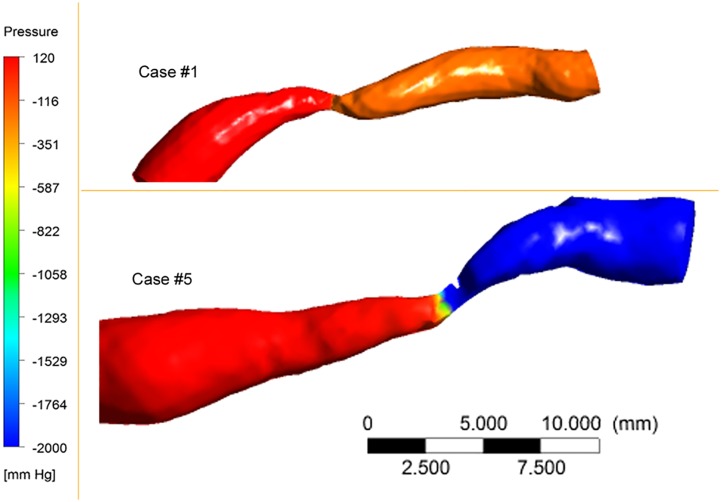
**Pressure maps of 80% MCA stenosis**. Proximal vessel diameters and lengths of stenosis differ between the cases. Hemodynamic severity of the two cases differed.

The reach of non-invasive testing can be enhanced with post-processing techniques: consider the power of computational fluid dynamic analyses of coronary artery or intracranial artery flow as imaged with CTA. The Determination of FFR by Anatomic Computed Tomographic Angiography (*DeFACTO*) investigators performed a multicenter diagnostic performance study comparing non-invasive and invasive FFR in the coronary arteries. Non-invasive FFR on CTA analyzed with computational fluid dynamics had an accuracy of 73% (95% CI, 67–78%) with a sensitivity of 90% (95% CI, 84–95%) and specificity of 54% (95% CI, 46–83%) (16). Preliminary studies have extended this approach to the intracranial circulation, as illustrated in Figure 4 (unpublished data).

## Data Archiving Will Assume a More Prominent Role

Post-processing techniques such as CFD are novel, but are continuously improved upon and replaced. Raw non-invasive digital data captured today could easily be stored and reanalyzed tomorrow given new software developments. Thus, a clinical trial network could consider the archiving of even routinely collected imaging data to keep pace with evolving imaging technology.

## Overly Simplistic Models Might be Avoided

Topographic heterogeneity may be important in many stroke situations, such as perfusion imaging and its attempts to distinguish ischemic core, penumbra, and adjacent hypoperfusion, for example (17, 18). Our trials also do not emphasize the importance of gathering and analyzing data that shed light on temporal heterogeneity, which may be equally revealing. Perfusion delays are increasingly analyzed in acute stroke, but in populations with intracranial atherosclerosis, these findings may be chronic, limiting the utility of CTP and PWI utility when they subsequently present with acute stroke syndromes. Coincident topographic and temporal heterogeneity may be important in collateral systems, such as the role played by CBV gradients in the penumbra of acute ischemia (17, 18).

Trials should promote quantification, distinguishing the mere presence of an abnormality from the measured degree of that abnormality, Consider that in acute stroke our concept of penumbra is still undergoing clarification after decades of research.

Software advances and practical limitations point to the need to reconsider our “gold standards.” While cardiologists put FFR on the map initially with pressure sensitive invasive wires, they have moved on to CT based CFD methods to compute fractional flow (16). Neurologists should recognize when an invasive approach is impractical and begin to work toward acceptance of advanced software techniques and non-invasive testing to assess patients.

## The Upcoming NINDS Stroke Trials Network Offers an Excellent Opportunity to Create a More Sustainable and Effective Research Model That Includes Imaging

Financial pressures on governmental sources of funded research may be relieved somewhat with a new approach to imaging. It is unlikely that every treatment trial will be paired with an ancillary imaging study. Collecting routine imaging data more consistently could bypass the time and direct costs required to acquire and collect more rigorously defined imaging data. Newly developed imaging techniques could be supported by industry, within the framework of a trial design, rather than paid for routinely prior to demonstration of benefit.

A greater emphasis on imaging leads to better imaging training, greater acceptance, and more consistent results with better ultimate translation from trials back into clinical practice. A new approach to assessing neuroimaging in clinical trials could support the creation of a central imaging library to function as an efficient repository of imaging data lesion characterization or atlasing. Novel software can be tested in the larger imaging datasets to emerge from this approach, with an emphasis on improving remote real time viewing and analysis. Larger datasets will stimulate the development of new statistics (imaging statisticians), computer vision, and informatics analysis techniques. Imaging files themselves as in the DICOM standard can serve a dual role as vehicles or repositories to contain/write key clinical data in the header information of each file.

In summary, a unique opportunity in the field of stroke now exists to leverage technology, improved collaborative research with colleagues focusing on the diverse nature of stroke around the globe, momentum of NIH initiatives in a broad-based stroke network and the revolutionary foresight of precision medicine that is now transforming other specialties.

## Conflict of Interest Statement

Edward Feldmann has no disclosures. David S. Liebeskind is a consultant to Stryker, Inc., and Covidien, Inc.
